# ‘2TM proteins’: an antigenically diverse superfamily with variable functions and export pathways

**DOI:** 10.7717/peerj.4757

**Published:** 2018-05-11

**Authors:** Jasweer Kaur, Rachna Hora

**Affiliations:** Department of Molecular Biology and Biochemistry, Guru Nanak Dev University, Amritsar, Punjab, India

**Keywords:** STEVOR, RIFIN, PfMC-2TM, Rosetting, Maurer’s clefts, Immune evasion, Protein trafficking

## Abstract

Malaria is a disease that affects millions of people annually. An intracellular habitat and lack of protein synthesizing machinery in erythrocytes pose numerous difficulties for survival of the human pathogen *Plasmodium falciparum*. The parasite refurbishes the infected red blood cell (iRBC) by synthesis and export of several proteins in an attempt to suffice its metabolic needs and evade the host immune response. Immune evasion is largely mediated by surface display of highly polymorphic protein families known as variable surface antigens. These include the two trans-membrane (2TM) superfamily constituted by multicopy repetitive interspersed family (RIFINs), subtelomeric variable open reading frame (STEVORs) and *Plasmodium falciparum* Maurer’s cleft two trans-membrane proteins present only in *P. falciparum* and some simian infecting *Plasmodium* species. Their hypervariable region flanked by 2TM domains exposed on the iRBC surface is believed to generate antigenic diversity. Though historically named “2TM superfamily,” several A-type RIFINs and some STEVORs assume one trans-membrane topology. RIFINs and STEVORs share varied functions in different parasite life cycle stages like rosetting, alteration of iRBC rigidity and immune evasion. Additionally, a member of the STEVOR family has been implicated in merozoite invasion. Differential expression of these families in laboratory strains and clinical isolates propose them to be important for host cell survival and defense. The role of RIFINs in modulation of host immune response and presence of protective antibodies against these surface exposed molecules in patient sera highlights them as attractive targets of antimalarial therapies and vaccines. 2TM proteins are *Plasmodium* export elements positive, and several of these are exported to the infected erythrocyte surface after exiting through the classical secretory pathway within parasites. Cleaved and modified proteins are trafficked after packaging in vesicles to reach Maurer’s clefts, while information regarding delivery to the iRBC surface is sparse. Expression and export timing of the RIFIN and *Plasmodium falciparum* erythrocyte membrane protein1 families correspond to each other. Here, we have compiled and comprehended detailed information regarding orthologues, domain architecture, surface topology, functions and trafficking of members of the “2TM superfamily.” Considering the large repertoire of proteins included in the 2TM superfamily and recent advances defining their function in malaria biology, a surge in research carried out on this important protein superfamily is likely.

## Introduction

Malaria remains as one of the major health burden worldwide. There were an estimated 212 million cases of malaria with 429,000 deaths in 2015. A total of 70% of these deaths occurred among children under five years of age (Organization, 2016). Apicomplexan pathogenic parasite *Plasmodium falciparum* is the most dangerous species causing human malaria. Clinical manifestation of this disease involves fever, headache, shaking chills, anemia and an enlarged spleen. Acute malaria may lead to secondary complications including coma, hypoglycemia, metabolic acidosis, renal failure and pulmonary edema (White & Ho, 1992). Mortality and morbidity associated with malaria is related to asexual stages of parasites present in red blood cells (RBCs). During this phase of growth, parasites reside within a parasitophorous vacuole (PV) and induce dramatic modifications of the host cell. These involve altered adhesive properties of the infected red blood cells (iRBCs), generation of knob-like structures on the cell surface, genesis of parasite induced membranous structures in the host cell, establishment of new pathways for nutrient export and import and display of antigenically diverse molecules on the host cell surface. A large repertoire of parasite proteins exported to the erythrocyte mediate these changes, where a subset of these are surface exposed and bind various host cell surface receptors. Ligand-receptor binding chiefly leads to “cytoadherence,” a key phenomenon in the pathophysiology of the disease (Ho & White, 1999). Cytoadherence involves binding of infected cells to vascular endothelium and their sequestration in the deep vasculature of various organs. Thus, cytoadhesion provides a survival advantage to parasite infected host cells by escaping the splenic clearance mechanism, host antibodies and the complement system (Cranston et al., 1984; Ho et al., 1990; Looareesuwan et al., 1987). Based on the presence of a conserved pentameric *Plasmodium* export elements/Host targeting (PEXEL/HT) motif, approximately 400 parasite proteins are predicted to be exported (Hiller et al., 2004; Marti et al., 2005); some of the exported proteins are PEXEL negative or contain a noncanonical PEXEL motif (Blisnick et al., 2000; Hawthorne et al., 2004; Spielmann et al., 2006; Spycher et al., 2003).

Malaria parasites are believed to evade the host immune response by expression of antigenically diverse multicopy protein families (collectively known as variable surface antigens (VSAs)) on the surface of infected host cells (Ferreira, da Silva Nunes & Wunderlich, 2004). VSAs include repetitive interspersed family (RIFIN), subtelomeric variable open reading frame (STEVOR), *Plasmodium falciparum* Maurer’s cleft 2 trans-membrane proteins (PfMC-2TM), surface-associated interspersed gene family proteins and *Plasmodium falciparum* erythrocyte membrane protein1 (PfEMP1) protein families (Baruch et al., 1995; Cheng et al., 1998; Ferreira, da Silva Nunes & Wunderlich, 2004; Gardner et al., 1998; Kyes et al., 1999; Leech et al., 1984; Petter et al., 2007; Smith et al., 1995; Su et al., 1995; Weber, 1988) (Table 1). Most genes encoding these families are present on the subtelomeric regions of chromosomes to generate hypervariability by recombination events (Kyes et al., 1999). Export of several of these VSAs occurs through parasite induced membranous structures in the iRBC cytoplasm known as “Maurer’s clefts” (MCs). MCs act as a sorting platform for protein trafficking, and are different from the tubulovesicular network (TVN) extending from the parasitophorous vacuole membrane (PVM) (Lanzer et al., 2006; Tilley et al., 2007; Wickert & Krohne, 2007).

**Table 1:** Variable surface antigens (VSAs) of *Plasmodium*, their receptors and functions.

Protein family	Genes encoding the protein families	Subtypes and chromosomal location	Host receptors	Functions
PfEMP1	*var* (*∼ 60*)	varA, varB/A (Telomeric and subtelomeric) varB (Telomeric and centromeric) varC, varB/C (centromeric) Lavstsen et al. (2003)	CD36, ICAM1, VCAM1, TSP, CR1q, P-selectin, E-selectin, EPCR, CSA, Heparan sulfate, IgM, α_2_ macroglobulin	Generation of antigenic diversity, immune evasion and cytoadherence
RIFIN	*rif* (*∼ 159*)	A-RIFINs and B-RIFINs Subtelomeric	Glycophorin A on red blood cells	Involvment in rosetting Goel et al. (2015), merozite invasion Mwakalinga et al. (2012), generation of antigenic diversity
STEVOR	*stevor* (*∼ 29*)	Subtelomeric	Glycophorin C on red blood cells	Rosetting, merozoite invasion Niang et al. (2014), and alteration of erythrocyte deformability Sanyal et al. (2012)
PfMC-2TM	*PfMC-2TM* (*∼ 13*)	Subtelomeric	Not determined	Probable role in formation of solute ion channels or providing anchoragment to surface exposed proteins Sam-Yellowe et al. (2004)
SURFIN	*surf* (*∼ 10*)	Subtelomeric	Not determined	Merozoite invasion Winter et al. (2005)

The PfEMP1 protein family encoded by *var* (∼60 genes) is central to cytoadherence and host immune evasion. These serve as ligands for various endothelial receptors like vascular cell adhesion molecule1, cluster of differentiation 36, intercellular adhesion molecule1, thrombospondin, P- selectin, E- selectin, endothelial protein C receptor and placental receptor chondroiton sulfate A (Beeson et al., 2000; Berendt et al., 1989; Cooke et al., 1994; Fried & Duffy, 1996; Ockenhouse et al., 1992; Senczuk et al., 2001; Turner et al., 2013). Constituent members lack an N-terminal signal sequence and the PEXEL motif. Numerous reports regarding characterization of PfEMP1 and molecules that assist its trafficking to the RBC membrane exist in the literature. A few of these reports propose vesicle mediated trafficking (Taraschi et al., 2001), while a majority of the reports suggest trafficking in the form of chaperone-associated soluble complexes upto MCs, (Knuepfer et al., 2005; Papakrivos, Newbold & Lingelbach, 2005) followed by vesicular transport from MCs to the iRBC surface (McMillan et al., 2013).

Biological roles and trafficking pathways of other VSAs, i.e., RIFINs, STEVORs and PfMC-2TM proteins constituting the 2TM superfamily are sparsely studied. Several 2TM superfamily proteins that were originally believed to carry 2TM domains on the basis of their predicted domain organization are now proven to be single pass proteins. Some members of RIFIN and STEVOR protein families have been shown to be responsible for rosetting and sequestration (Goel et al., 2015; Niang et al., 2014), while the role of PfMC-2TM proteins in parasite biology is not clear. However, trans-membrane (TM) regions of these and other 2TM superfamily members were proposed to provide membrane spanning anchors for knobs to facilitate display of PfEMP1, or formulate solute ion channels (Lavazec, Sanyal & Templeton, 2006). This review collates and comprehends existing information on the domain organization and membrane topology of different families of the 2TM superfamily, diverse roles played by them, membrane topology, their localization at different stages of the parasite life cycle and their trafficking pathways. The export mechanism of some 2TM superfamily members is partly defined, which occurs independently of PfEMP1.

## Survey Methodology

**Figure 1:**
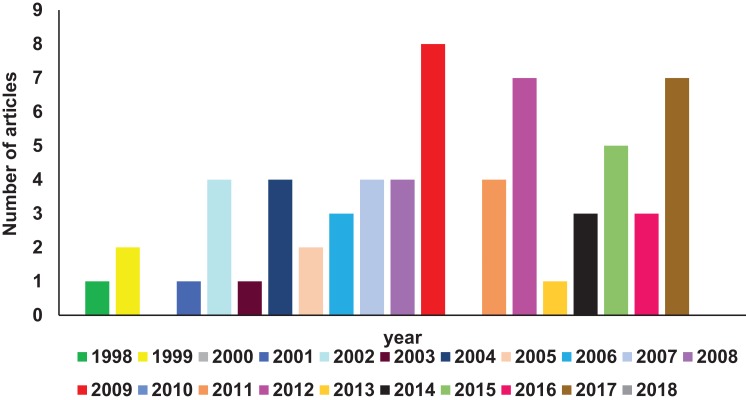
Diagrammatic representation of year-wise reports on 2TM superfamily found in Pubmed database.

A comprehensive search of Pubmed literature database (Motschall & Falck-Ytter, 2005) (key words: RIFIN; STEVOR; PfMC-2TM, 2TM superfamily) was performed to find studies related to the 2TM superfamily. Reports relevant to the scope of this article have been comprehended and reviewed here. Figure 1 shows a year-wise diagrammatic representation of the number of articles published on the 2TM superfamily or its constituent families. Since their discovery in 1998, a total of 56 articles were found almost uniformly spread over the last 15 years. Members of these clonally variant protein families have diverse temporal and spatial expression patterns, lack any known unified role and have a poorly defined significance in parasite biology. Despite their vast representation in the *Plasmodium* genome, limited information on these proteins is available possibly owing to the discovery of the PfEMP1 family in 1995 (Smith et al., 1995; Su et al., 1995) followed by its rapid functional and structural characterization (Singh et al., 2006; Tolia et al., 2005). The PfEMP1 family encoded by *var* genes are key pathogenic molecules involved in cytoadherence that attracted most attention of researchers while small variant antigens were largely ignored. Recent research on RIFINs since 2015 has highlighted their role in rosetting (Goel et al., 2015), immune evasion and suppression of host immune effector components (Saito et al., 2017). These important discoveries are likely to be the initial milestones in understanding the unexplored functions of this superfamily. Localization on all 14 chromosomes (Gardner et al., 2002) and lack of efficient methods to knock-out or knock-down entire gene families in *Plasmodium* species (McRobert & McConkey, 2002; Prommana et al., 2013) form major bottlenecks in identifying the functional relevance of the 2TM superfamily. Additional roadblocks include absence of orthologues in rodent infecting species and existing simian models in order to study these families.

## Orthologues of 2TM Superfamily in other *Plasmodium* Species

**Figure 2:**
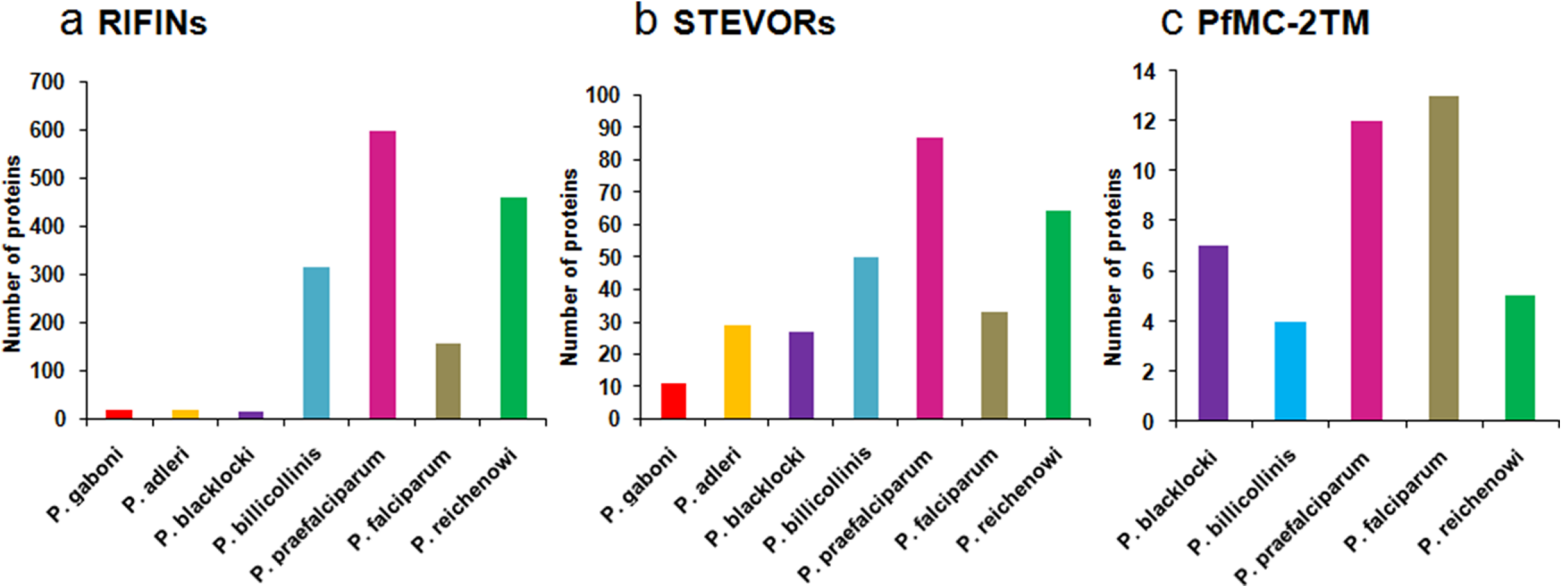
Distribution of RIFIN, STEVOR and PfMC-2TM orthologues in *Plasmodium* species. (A) RIFINs; (B) STEVORs; (C) PfMC-2TM.

Multicopy subtelomeric 2TM superfamily members are also present in other *Plasmodium* species. BLASTP analysis on Plasmodb.org revealed orthologues of RIFINs and STEVORs in gorilla infecting species *P. adleri, P. blacklocki and P. praefalciparum* and chimpanzee infecting *P. gaboni, P. billicollinis* and *P. reichenowi*. PfMC-2TM proteins are also found to have orthologues in simian species *P. billicollinis, P. blacklocki, P. praefalciparum* and *P. reichenowi*. None of the rodent, avian or other human infecting species had proteins that shared significant homology with the 2TM superfamily members. Figure 2 shows a bar diagram depicting the number of (a) RIFIN, (b) STEVOR and (c) PfMC-2TM proteins in different *Plasmodium* species.

Most of the identified simian orthologues of RIFINs and STEVORs were annotated as “*Plasmodium* interspersed repeats” (PIR). The “PIR” superfamily includes members of RIFINs/STEVORs, VIR (*P. vivax*), KIR (*P. knowlesi*), YIR (*P. yoelli*), BIR (*P. berghei*) and CIR (*P. chabaudi*). Detailed information regarding the PIR superfamily has been reviewed by Jemmely, Niang & Preiser (2010). Though most PIRs lack sequence similarity with the 2TM proteins of *P. falciparum*, they share secondary structural similarities, a common but distant evolutionary relationship and therefore are likely to be functionally convergent (Janssen et al., 2004). Second introns of *rif* and *kir/yir/bir* families share significant sequence identity (∼72%) suggestive of their common ancestry. Also, the rif and kir/yir/bir genes may follow a common regulation of gene expression since RNAs arising from introns of *Plasmodial* genes have been previously reported to be involved in such processes (Amit-Avraham et al., 2015; Mishra, Kumar & Sharma, 2009).

## Domain Organization and Topology of 2TM

**Figure 3:**
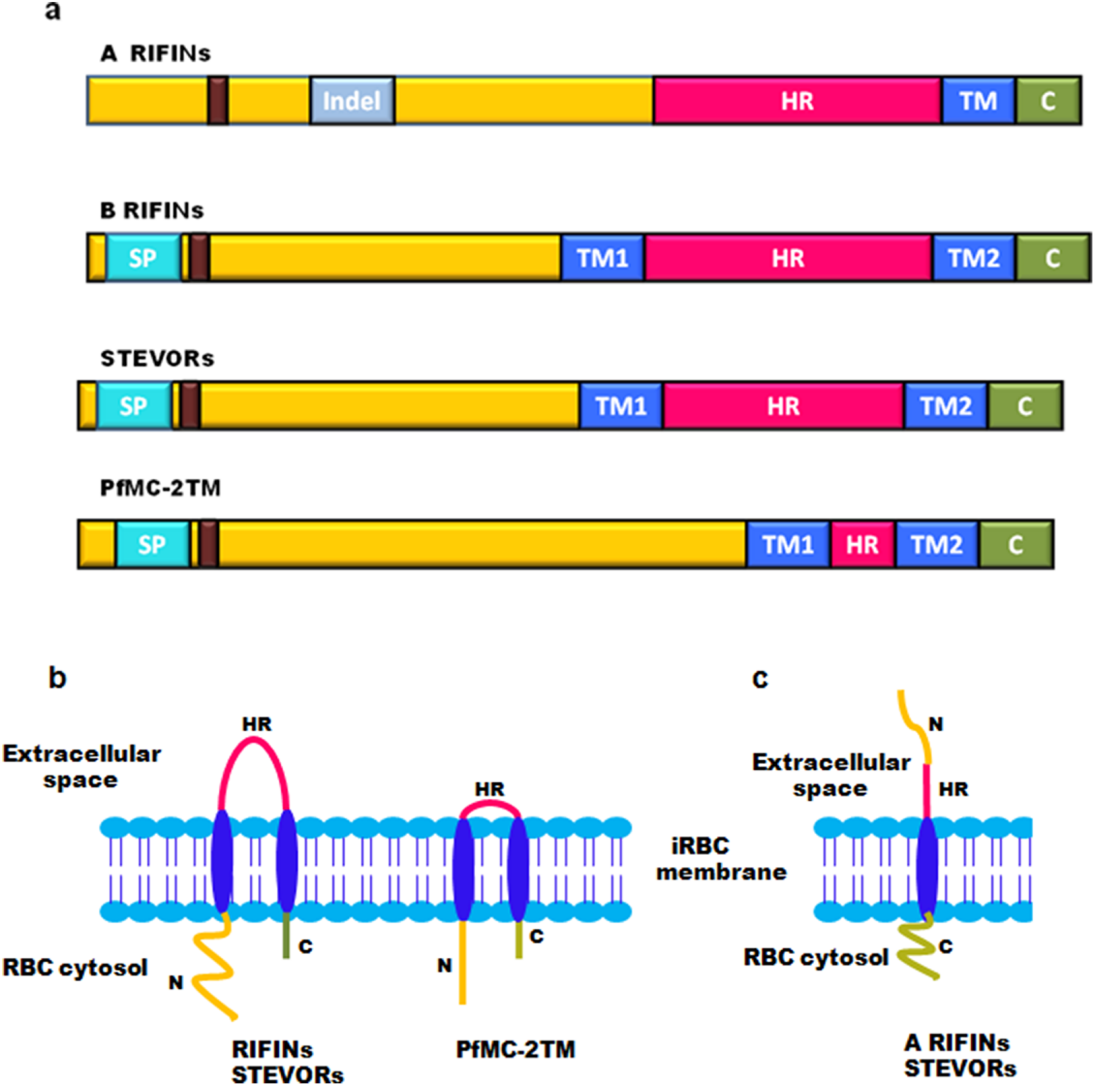
Domain organization and membrane topology of 2TM superfamily. (A) Domain organization of A RIFINs, B RIFINs, STEVORs and PfMC-2TM (B) Two trans-membrane topology model of RIFINs, STEVORs and PFMC-2TM (C) One trans-membrane topology model of A-type RIFINs and STEVORs. SP, signal peptide; Indel, insertion deletion sequence; TM, trans-membrane region; HR, hypervariable loop region; C, conserved C-teminus; N, N-terminal semiconserved region and the PEXEL motif (brown box).

On the basis of TM region prediction, 2TM superfamily contains over 200 genes encoding RIFINs, STEVORs and PfMC-2TM proteins. Sequence alignment studies show that most of these protein families consist of a semiconserved N-terminal region carrying a signal peptide, two conserved TM domains flanking a hypervariable loop, and highly conserved C-terminal residues carrying a lysine-rich C-terminal tail present after the second TM domain (Fig. 3A) (Sam-Yellowe et al., 2004). The hypervariable loop is a surface exposed region displayed on infected erythrocytes, which has a proposed role in generating antigenic diversity. All Pf2TM proteins carry a PEXEL/HT motif that facilitates trafficking of proteins across the PVM (Hiller et al., 2004; Marti et al., 2004). The RIFIN family has two subtypes: A and B based on the presence or absence of a defined 25 amino acid Indel (Insertion/deletion) sequence (Joannin et al., 2008). As demonstrated by signal peptide and TM region prediction using signalIP 3.0 and con pred II, respectively, most of the B-type RIFINs from Pf3D7 carry a signal peptide and 2TM domains, while most A-type RIFINs lack a signal peptide and carry only one trans-membrane (1TM) domain on their C-terminus (Bultrini et al., 2009). A different domain organization for A and B-type RIFINs is likely to impact their structure and function.

Membrane topology of surface exposed proteins bears a very important implication on their function since this determines their domain accessibility to host immune effectors. The initially proposed “2TM” topology model for the Pf2TM superfamily (Fig. 3B) is now challenged by a “1TM” model particularly for some STEVORs and most A-type RIFINs. Evidence includes in silico secondary structure prediction for RIFINs (Bultrini et al., 2009) and experimental data for STEVORs and RIFINS (Bachmann et al., 2015). The 1TM model suggests these proteins to carry a single TM domain so that their semiconserved N-terminal region and hypervariable loop are exposed on the erythrocyte surface, while their conserved C-terminal domain points within the cytosol (Fig. 3C). Therefore, during export via these structures, their N-terminal stretch and the loop must both jut into the lumen of MCs. Immunofluorescence assays (IFAs), immunoelectron microscopy (IEM) and cellular fractionation studies on infected erythrocytes using protein specific antisera (RIFIN (RIF40.2 and RIF44), STEVORs (PF3D7_1254100, PF3D7_0115400, PF3D7_0300400 and PF3D7_1300900) and PfMC-2TM (PF3D7_0631400SC and PF3D7_0631400CT)) have experimentally pinned down membrane localization and orientation of these proteins (Bachmann et al., 2015). Cellular fractionation studies detected these proteins in the pellet of hypotonically and saponin lysed iRBCs, indicating these to be membrane associated. Trypsin treatment of *P. falciparum* infected erythrocytes prior to lysis led to a significant reduction in detection of RIFINs (RIF40.2 and RIF50), while a slight reduction was observed for STEVORs (PF3D7_0115400 and PF3D7_0300400). Additionally, protein specific antisera against the semiconserved N-terminal region of these proteins failed to detect any band post trypsin treatment indicating the surface exposure of this domain. In contrast, antisera against PfMC-2TM proteins for both semiconserved and C-terminal domains detected neither a size shift nor a change in signal intensity post trypsin treatment, suggesting a 2TM topology for these proteins. These results were also confirmed by differential permeabilization and trypsinization of iRBCs, providing clear evidence for 1TM topology of most A-RIFINs and some variants of STEVORs. However, STEVOR proteins need further experimentation to completely understand their TM orientation.

Contrary to their nomenclature, members of the PfMC-2TM family are more abundantly localized at the iRBC surface as compared to MCs. Membrane extraction studies on hypotonically lysed *P. falciparum* infected erythrocytes indicate that RIFINs and STEVORs are TM proteins that reside in the detergent resistant areas of the membrane. However, PfMC-2TM proteins span the membrane outside the detergent resistant regions. Interestingly, STEVORs associate with the membrane by protein–protein interactions, while PfMC-2TM proteins are integrated by protein–lipid interactions (Bachmann et al., 2015).

## Rifin Proteins

Subtelomeric multicopy gene family, *“Repetitive interspersed family* (rif)” encodes RIFINs, and represents the largest group (150–200 copies) of VSAs identified in *P. falciparum* (Cheng et al., 1998; Fernandez et al., 1999; Kyes et al., 1999). Genes encoding RIFINs are clustered with those of *var* and *stevor* (Gardner et al., 2002). As discussed, RIFIN proteins are classified into two subgroups: A and B type (Joannin et al., 2008). The A-type proteins are generally 25 amino acids longer than the B-type variants (∼350 and ∼330 amino acids, respectively). This short and defined peptidic stretch is present in the semiconserved region of A-type RIFINs downstream of the PEXEL motif. Additionally, A-type RIFINs carry a total of ten highly conserved cysteine residues, while B-type variants carry only six; five of these cysteines are common amongst both subtypes. RIFINs are transcribed 12 hours post invasion (hpi), and appear on the erythrocyte surface at 16–20 hpi, coherently with PfEMP1 (Kyes et al., 1999). Localization studies using IFAs on asexual stage parasites showed simultaneous expression of both categories of RIFINs, where A-type were displayed on infected erythrocyte membrane as discrete punctate structures, while B type showed fluorescence distributed within the parasite (Petter et al., 2007). Their IFA patterns dictate A-type RIFINs to be exported *via* Maurer’s clefts to the erythrocyte membrane, while B-type RIFINs seem to accumulate mainly inside the parasite.

### Expression of RIFINs at different stages of parasite life cycle

Proteome and transcriptome wide analyses have indicated that RIFIN expression occurs not only in asexual intraerythrocytic stages, but also in gametocytes as well as invasive parasitic stages such as merozoites and sporozoites (Table 2). Transcriptome studies on differentiating gametocytes show continuous transcription of RIFINs with differential expression of variants (Wang et al., 2010). For some members, expression is highest at gametocyte stages III and IV, and declines at stage V. r*if* transcript PF3D7_0900500 of *P. falciparum* 3D7/NF54 shows highest expression during gametocyte stages II and III, while B type *rif* PF3D7_1300600 dominates at gametocyte V and sporozoites stages. Western blot and IFA studies on sexual and asexual stage parasites using anti PF3D7_1300600 antibodies support the results obtained from transcript analysis (Mwakalinga et al., 2012). Interestingly, PF3D7_1300600 is the only B-type RIFIN that was found to be exported to the surface of invading merozoites, suggesting its role in sensing or binding to new RBCs.

**Table 2:** Stage specific proteome wide expression analysis of RIFIN members using mass spectrometry (Florens et al., 2002).

Parasite life cycle stage	RIFIN proteins
Sporozoites	PF3D7_0200700, PF3D7_0223400, PF3D7_0300700, PF3D7_0400300, PF3D7_0401200, PF3D7_00401300, PF3D7_0617700, PF3D7_0732700, PF3D7_0900200, PF3D7_0937400, PF3D7_01040800, PF3D7_1100400, PF3D7_1150000, PF3D7_1200300, PF3D7_1254200, PF3D7_0421200, PF3D7_0200700, PF3D7_0223400, PF3D7_1400300, PF3D7_1400200, PF3D7_1400400, PF3D7_1040800, PF3D7_1100400, PF3D7_1150000, PF3D7_1300600, PF3D7_0617700, PF3D7_1040400
Sporozoites, gametocytes	PF3D7_0114700, PF3D7_0115200
Sporozoites, merozoites, gametocytes	PF3D7_900300
Merozoites	PF3D7_0901100, PF3D7_1000500
Gametocytes	PF3D7_0222700, PF3D7_0425700, PF3D7_0608600, PF3D7_901000, PF3D7_0632300
Sporozoites, trophozoites, merozoites, gametocyte	PF3D7_0900500
Intraerythrocytic stages	PF3D7_0401600, PF3D7_0300200, PF3D7_1101300, PF3D7_0324800

Immunofluorescence studies using antiA-rif40 and antiB-rifΔNC (PF3D7_0901000) antibodies show that distribution of A and B type RIFINS during gametocyte differentiation is the same as that for asexual stages, i.e., A type RIFINs show a crescent shaped punctate pattern on the erythrocyte membrane, while B-type RIFINS are present within the parasite (Petter, Bonow & Klinkert, 2008). During the merozoite stage, A-type RIFINs are localized at the apical tip, while B-type are distributed in the cytosol with both being coexpressed in same parasite (Petter et al., 2007). Colocalization of rif-40 with PfSBP1 (MC resident protein) indicates that these proteins occupy the same subcellular compartment in gametocytes. The staining pattern of Pfg27, a cytoplasmic gametocyte protein dispensable for maintenance of gametocytogenesis poorly overlaps with A-type RIFIN “rif40,” while complete overlapping was observed with B-type RIFIN rifΔNC (Petter, Bonow & Klinkert, 2008). The differential localization of A and B-type RIFINS is indicative of their varied roles in parasite biology. Expression of these proteins at multiple stages of parasite life cycle proposes them to be significant in parasite biology, while functions of very few RIFINs have been determined.

### Role in immune evasion and protection

An involvement in antigenic variation is strongly supported by the presence of a hypervariable region and the existence of a large repertoire of more than 150 *rif* genes in the genome of a single parasite. Additionally, the significance of RIFIN proteins in immune protection is evident from the correlation between the level of anti-RIFIN antibodies in the plasma of infected individuals and clearance of parasite from circulation (Abdel-Latif et al., 2003). To correlate levels of anti-RIFIN antibodies with parasite clearance rate, recombinant RIFIN antigens (rif29, rif44, rif4 and rif50) were used in this study to detect antibody levels in serum samples of infected individuals. High antibody titers led to rapid clearance of circulating parasites in patients, and a doubling of anti-RIFIN antibodies significantly reduced clearance time. However, presence of antibodies did not prevent reinfection. High level of RIFIN antibodies was correlated with suppression of malaria symptoms in asymptomatic children. Interestingly, RT-PCR based transcript analysis on clinical isolates of *P. falciparum* showed significantly higher expression of A-type RIFINs while levels of B-type matched those from laboratory strain 3D7, suggestive of a role for A-type RIFINs in survival within the human host (Bachmann et al., 2012). Immunoblot analysis of saponin lysed clinical samples using anti-RIFIN antibodies further validated these observations, where significantly higher expression levels of A-type RIFINs were observed as compared to in vitro 3D7 cultures.

Further, broadly reactive antibodies detected in sera of malaria infected individuals agglutinate and opsonize the infected erythrocytes. Sequence analysis of these antibodies reveals the presence of a 98 amino acid stretch corresponding to the collagen binding domain of leucocyte-associated immunoglobulin-like receptor1 (LAIR1) inserted between the V and DJ fragments by interchromosomal transposition (Tan et al., 2016). However, three mutated residues disallow it to interact with its natural partner collagen. These antibodies could immunoprecipitate RIFINs PF3D7_1400600, PF3D7_1040300 and PF3D7_0100400 from the ghost fraction of agglutinated iRBCs. They also selectively stained the Chinese hamster ovary cells transfected with RIFINs PF3D7_1400600 and PF3D7_1040300 but not PF3D7_0100400, PF3D7_0100200 and PF3D7_1100500. Presence of such diversified circulating antibodies is suggestive of their role in promoting phagocytosis, iRBC destruction and hence parasite clearance.

Repetitive interspersed family participate in immune evasion of infected erythrocytes and host immune suppression by interacting with leucocyte immunoglobulin-like receptor B1(LILRB1), which is an inhibitory receptor present on T cells, B cells, NK cells and monocytes (Saito et al., 2017). LILRB1 binds parasitized erythrocytes from clinical isolates and different laboratory strains of *P. falciparum*, and coimmunoprecipitates RIFIN peptides from *P. falciparum* infected cell lysates. iRBCs transfected with RIFINs PF3D7_1254800 and PF3D7_0223100 bind strongly to LILRB1and inhibit B cell mediated IgM production and NK cell activation. Therefore, these RIFINs downregulate host immune cells and reduce antimalarial immunity.

### Role in rosetting

Rosetting is a phenomenon of spontaneous binding of infected RBCs to uninfected RBCs. This can provide a growth advantage to parasites by generating a favorable environment for invading merozoites, and in evasion of iRBCs or newly released merozoites from the host immune system (Deans & Rowe, 2006; Wahlgren et al., 1989). Several scientific reports have been published regarding rosette formation and its implications on disease severity (Carlson et al., 1990; Kaul et al., 1991; Rowe et al., 1995; Wahlgren et al., 1992). Considering the significance of rosetting, the molecular contributors in the process have attracted the scientific community. RIFINs bind glycophorin A on O blood group RBCs to form small rosettes, while their binding to N-acetylgalactosamine on blood group A RBCs results in formation of large rosettes independently of PfEMP1 (Goel et al., 2015). Yam et al. (2017) have recently reviewed the detailed role of RIFIN, STEVOR and PfEMP1 families in rosetting.

## Stevor Proteins

Subtelomeric variable open reading frame protein family (Mol. Wt. 30–40 kDa) encoded by *stevor* genes is the third largest family of VSAs after RIFINs and VARs (Gardner et al., 2002). Genes encoding STEVORs are located subtelomerically on all the 14 chromosomes of *P. falciparum. stevors* are a multicopy gene family comprised of approximately 30–40 members in different laboratory strains of *P. falciparum* (Cheng et al., 1998). Multiple *stevor* transcripts are detected in a single parasite, and their expression is clonally variant. Microarray and RT-PCR studies show that PF3D7_0901600 transcript is expressed in clone 5.2, PF3D7_1040200, PF3D7_0101800 and PF3D7_0500600 in clone 3.2c and PF3D7_1040200, PF3D7_0101800, PF3D7_0500600 and PF3D7_1479500 in clone 5A (Niang, Yan Yam & Preiser, 2009). *stevor* transcription is maximum during mid trophozoite stage of asexual parasite development (at approximately 28 hpi) (Kaviratne et al., 2002). These proteins first localize to the iRBC cytosol, and are then trafficked to the erythrocyte membrane *via* MCs. MC localization of these proteins was confirmed by colocalization with PfSBP1 and *Plasmodium falciparum* erythrocyte membrane protein3 (PfEMP3). Indirect IFA, live IFA, western blotting and cell agglutination assays using anti-S1 and anti-S2 antibodies against the N-terminal semiconserved regions of two different STEVORs (PF3D7_1040200 and PF3D7_0617600) clearly show trypsin sensitive surface display of these proteins (Niang, Yan Yam & Preiser, 2009). Protein extraction studies of saponin lysed iRBCs using bicarbonate buffer demonstrate these as integral membrane proteins. Surface display of STEVOR proteins on infected erythrocytes further adds complexity to iRBC immunogenicity, and may help parasites in immune evasion. STEVORs are considered proteins having diverse functionality due to their stage dependent expression patterns and involvement in various processes of parasite life cycle (Blythe, Surentheran & Preiser, 2004). Their expression pattern at different parasite life cycle stages and their functions are discussed below.

### Differential expression of STEVORs in parasite life cycle

Subtelomeric variable open reading frames are expressed at both asexual and sexual stages of parasite life cycle including trophozoites, schizonts, merozoites, gametocytes and sporozoites. RT-PCR studies on STEVOR transcripts show differential abundance at trophozoite and early gametocyte stages (Sharp et al., 2006). While PF3D7_1040200, PF3D7_0617600 and PF3D7_0401500 transcripts are abundant at early gametocyte (stage II) stage, trophozoite dominant PF3D7_0901600 is downregulated. Also, these proteins show distinct localization patterns in asexual and sexual stages. Unlike asexual stages, STEVORs do not colocalize with PfSBP1 (MC resident protein) in gametocytes and are trafficked through a MC independent pathway (McRobert et al., 2004). At sporozoite stage, these proteins localize to discrete foci in a pattern different from gametocytes and asexual stage parasites. This variation is indicative of diverse functions performed by this protein family at different parasitic stages. Apart from these localization studies on STEVORs, mass spectrometric data shows expression of PF3D7_0617600 and PF3D7_1254100 at schizont and sporozoite stages, respectively (Lasonder et al., 2012; Lasonder et al., 2008). No protein level expression data is available for any of the other members of this family.

### Role in merozoite invasion

In the merozoite stage of parasite life cycle, STEVORs are colocalized on the surface with Merozoite surface protein1 (PfMSP1) throughout the invasion process (Niang et al., 2014). Role of STEVORs in merozoite invasion is validated by the inhibitory effect of anti-STEVOR antibodies (antiS1 and antiS2) on different parasite clones (5A, 3.2C, 5.2A, 5B and A4). Antibodies displayed invasion inhibition for clone 5A in a dose dependent manner equivalent to that of anti-MSP1 antibodies. Effect of antiS1 and antiS2 antibodies occurred in a variant specific manner; live video microscopy during merozoite invasion clarified that anti-STEVOR antibodies affect the initial steps of merozoite adhesion to the erythrocytes. Colocalization of STEVORs with PfMSP1 and invasion inhibitory effect of anti-STEVOR antibodies together signify the role of STEVOR proteins in merozoite invasion, an important step for parasite propagation.

### Role in rosetting

The PfEMP1 family has been known to be involved in rosetting for a long time (Rowe et al., 1997), while STEVORs have more recently been implicated in the process. This role was experimentally proven by western blot analysis of rosetting positive parasite clones 5A-R+, 5.2A-R+ and 3.2C-R+ using STEVOR specific antiS1 and antiS2 antibodies (Niang et al., 2014). These clones showed increased expression of STEVORs as compared to rosetting negative clones. Pre-incubation of 5A-R+ clone infected RBCs with anti STEVOR antibodies reduced the rate of rosette formation by 50% as compared to untreated cells. The A4 parasite clone, which neither transcribes nor expresses STEVORs, showed surface expression of STEVORs on transfection with *stevor* gene (PF3D7_1040200), and formed rosettes, which could be disrupted by antiS1 antibodies. The N-terminal semiconserved regions of STEVORs PF3D7_1040200, PF3D7_0617600 and PF3D7_0102100 were seen to have roles in rosette formation, as evident from efficient binding of their recombinant constructs SC1, SC2 and SC3, respectively to RBCs.

3D7ΔMAHRP1-iRBCs do not display PfEMP1 on their surface, while STEVORs are normally trafficked in this line. Despite the absence of surface PfEMP1, 3D7ΔMAHRP1 iRBCs are able to rosette correctly. However, addition of anti-STEVOR antibodies disrupts rosette formation, strongly supporting the role of STEVORs in the process. Additionally, monoclonal anti-glycophorin C antibody blocks rosette formation, and soluble glycophorin C competitively binds with STEVORs and inhibits rosette formation in a concentration dependent manner. Together, these observations demonstrate how STEVORs bind to chymotrypsin resistant glycophorin C receptors on RBCs and facilitate rosetting independently of PfEMP1.

### Role in alteration of RBC rigidity

Increased rigidity of *P. falciparum* iRBCs leads to decrease in the cell deformability that offers increased haemodynamic resistance to blood cells in vascular capillaries. This, together with cytoadhesion, culminates in occlusion of deep blood vessels and deprivation of various tissues from blood and nutrient supply. Therefore, the parasites form a comfortable niche within the host, and evade immune response. Several proteins like Knob-associated histidine rich protein, PfEMP3 (Glenister et al., 2002) and *Plasmodium falciparum* ring infected erythrocyte surface antigen (Mills et al., 2007) have been known to be key players in reducing erythrocyte deformability. Recent studies suggest a similar role for STEVOR proteins in the process. Experimental evidence includes an increased retention time on beads of microspheres for clones B3 and H4 (express two or more STEVOR genes) in contrast to clones B3B1 (lack STEVOR expression) and A12 and E10 (very low STEVOR expression) (Sanyal et al., 2012). A transgenic parasite line overexpressing STEVOR (PF3D7_0631900) also showed increased retention time; consistent results were obtained by ektacytometric analysis. Deformability of gametocyte infected RBCs is regulated by phosphorylation of the cytoplasmic domain of STEVOR PF3D7_0631900 (Naissant et al., 2016). Additionally, the strength of interaction between the cytoplasmic tail of STEVORs and host cytoskeletal ankyrin is altered by STEVOR phosphorylation through protein kinase A. However, the relation between STEVOR phosphorylation and iRBC stiffness during asexual stages has not been determined.

### Role in immune evasion

Since in vitro cultured parasites do not face any immune selection pressure, molecules that show enhanced expression in clinical isolates are likely to contribute to immune evasion and parasite survival in the human host (Blythe et al., 2008). STEVOR detection frequency was significantly higher (>90%) on iRBCs from fresh field samples as compared to culture-adapted parasites (<30%) when studied using IFAs. This highlights the probable role that STEVORs may play in immune evasion and in vivo parasite survival.

## PfMC-2TM Proteins

A novel subtelomeric gene family encodes PfMC-2TM (Gardner et al., 2002). This family consists of 13 members (Mol. wt. ∼25 kDa) that carry a conserved pair of cysteine residues. PfMC-2TM family members show sequence conservation across their N-terminus and carry a PEXEL motif, 2TM domains and a conserved C-terminus (Sam-Yellowe et al., 2004). A variable stretch of ∼3–9 amino acid residues between the 2TM domains is highly polymorphic, and diversity within this region is believed to generate antigenic variation. One of the conserved TM domain carries proline residues that may induce kinked conformation feature of ion channels, receptors and gates. RT-PCR based transcript studies suggest members of this family to be expressed in a clonally variant fashion (Lavazec, Sanyal & Templeton, 2007). Mass spectrometry based proteome analysis reveals the stage specific expression profile of PfMC-2TM members (Table 3) (Bowyer et al., 2011; Florens et al., 2002; Lasonder et al., 2012; Oehring et al., 2012; Pease et al., 2013). Immunofluorescence studies show that PF3D7_0114100 colocalizes with ring exported protein (REX1) and PfEMP1, proposing it to share its function with REX1 as an anchor for the developing MCs to erythrocyte cytoplasm, and as a chaperone to assist PfEMP1 trafficking to MCs (Tsarukyanova et al., 2009). IEM shows that these proteins are also located in the PV/PVM. Extraction profiles of iRBCs to detect PfMC-2TM proteins show characteristics of integral membrane proteins that are Triton X-100 soluble, and IFAs reveal that these are more abundantly localized to erythrocyte membrane rather than MCs (Bachmann et al., 2015). Therefore, these seem to be integrated in the erythrocyte membrane by protein–lipid interactions. However, the exact biological role for any member of this family has not been elucidated.

**Table 3:** Stage specific proteome wide expression analysis of PfMC-2TM members using mass spectrometry.

Parasite life cycle stage	PfMC-2TM Protein ID
Schizonts and trophozoites	PF3D7_0701600,PF3D7_1100800, PF3D7_0101300, PF3D7_0221500
Trophozoites	PF3D7_0700800, PF3D7_1101700
Ring, schizont, trophozoites	PF3D7_1039700, PF3D7_0324100
Ring, trophozoites	PF3D_0601200, PF3D7_0222100
Sporozoites	PF3D7_0631400
Schizonts	PF3D7_0114100
Not determined	PF3D7_0713100

**Note:**

Bowyer et al. (2011), Florens et al. (2002), Lasonder et al. (2012), Lasonder et al. (2008), Oehring et al. (2012), and Pease et al. (2013).

## Trafficking of “2TM” Superfamily Members to the Erythrocyte Membrane

Lack of endogenous protein synthesis and translocation machinery poses a challenge for parasites to make their home habitable, which is resolved by generation of such components on their own (Tilley et al., 2007). Parasites reside within a self constructed PV inside the host cell, and export a diverse repertoire of proteins including several VSAs to the erythrocyte membrane. They also create novel membranous structures called MCs, which act as a sorting platform during protein trafficking. These subcellular entities further add complexity to the process of protein translocation since exported parasite proteins need to cross several membranes before they reach their final destination. Initial studies showed that STEVORs and PfMC-2TM proteins localize to MCs (Kaviratne et al., 2002; Przyborski et al., 2005a; Sam-Yellowe et al., 2004). However, now it is generally accepted that A-type RIFINs, STEVORs and PfMC-2TM members are displayed on the surface of iRBCs (Bachmann et al., 2015). After translation within the parasite, these proteins pass through various membranous structures including parasite endoplasmic reticulum (ER), parasite plasma membrane (PPM), PVM, MCs and finally infected RBC plasma membrane. Translocation across these membranes requires a minimum of three defined signals including (a) a N-terminal signal sequence (for entry into the secretory pathway) (b) HT/PEXEL motif (for transport across the PVM) (c) TM domains within the primary exported protein (for insertion into membranes) (Przyborski et al., 2005b). All RIFINs, STEVORs and PfMC-2TM proteins carry these three signals required for protein transport (Cheng et al., 1998; Sam-Yellowe et al., 2004), though B-type RIFINs reside within the parasite despite the presence of a PEXEL motif (Petter et al., 2007). Interplay of several molecular players occurs at each step of translocation, though the exact export pathway remains to be elucidated. Here, we have put together the existing information pertaining to trafficking of STEVORs and RIFINs for a comprehensive understanding of the process.

### Entry into the secretory pathway

*Plasmepsin V mediated cleavage of proteins:* Approximately 8% of *P. falciparum* proteins carry a PEXEL/HT motif. RIFINs and STEVORs possess a canonical PEXEL sequence at their N-terminus, and are therefore trafficked through a PEXEL dependent pathway (Hiller et al., 2004; Marti et al., 2004). This is a conserved motif carrying RXLXD/E/Q residues that serve as a substrate for an ER-resident protease, “Plasmepsin V” that cleaves after leucine (RXL↓). The newly generated N-terminus is acetylated, followed by export of processed proteins to the PV. This enzymatic cleavage of RIFINs and STEVORs was experimentally confirmed by incubation of PEXEL carrying synthetic peptides of RIFIN (PF3D7_0115300) and STEVOR (PF3D7_0300400) with Plasmepsin V agarose, and analysis by RP-HPLC and LC-MS/MS (Boddey et al., 2013). The Plasmepsin V substrate specificity is exquisitely defined; A single point mutation at position 1 (arginine) or postion 3 (leucine) completely abrogates the cleavage and protein trafficking. A study by Bhattacharjee et al. (2012) showed that newly synthesized proteins bind ER phospholipid “phosphatidyl inositol-3-phosphate (PI(3)P)” before plasmepsin V mediated cleavage, and considered it as a generalized property of the malaria exportome. Lipid sedimentation assays using PI(3)P coated vesicles also confirmed the binding. Surface plasmon resonance studies showed that green fluorescent protein linked RTLSE motif of RIFIN (PF3D7_0401600) and RLLAQ motif of STEVOR (PF3D7_0101800) bind with high affinity to PI(3)P, and binding affinity is reduced 20-folds upon replacement of RXLXE/D/Q with AxLXE/D/Q. However, these findings were strongly challenged by Boddey et al. (2016), who experimentally showed that PI(3)P is present on the food vacuole membrane, and not on the inner leaflet of ER membrane as proposed by Bhattacharjee et al. (2012). They found binding of PI(3)P to the PEXEL motif of exported proteins to be irreproducible. Also, the spatial position of PEXEL motif on the protein sequence must be conserved for cotranslational processing and export.

### Transport across PPM and PVM

**Figure 4:**
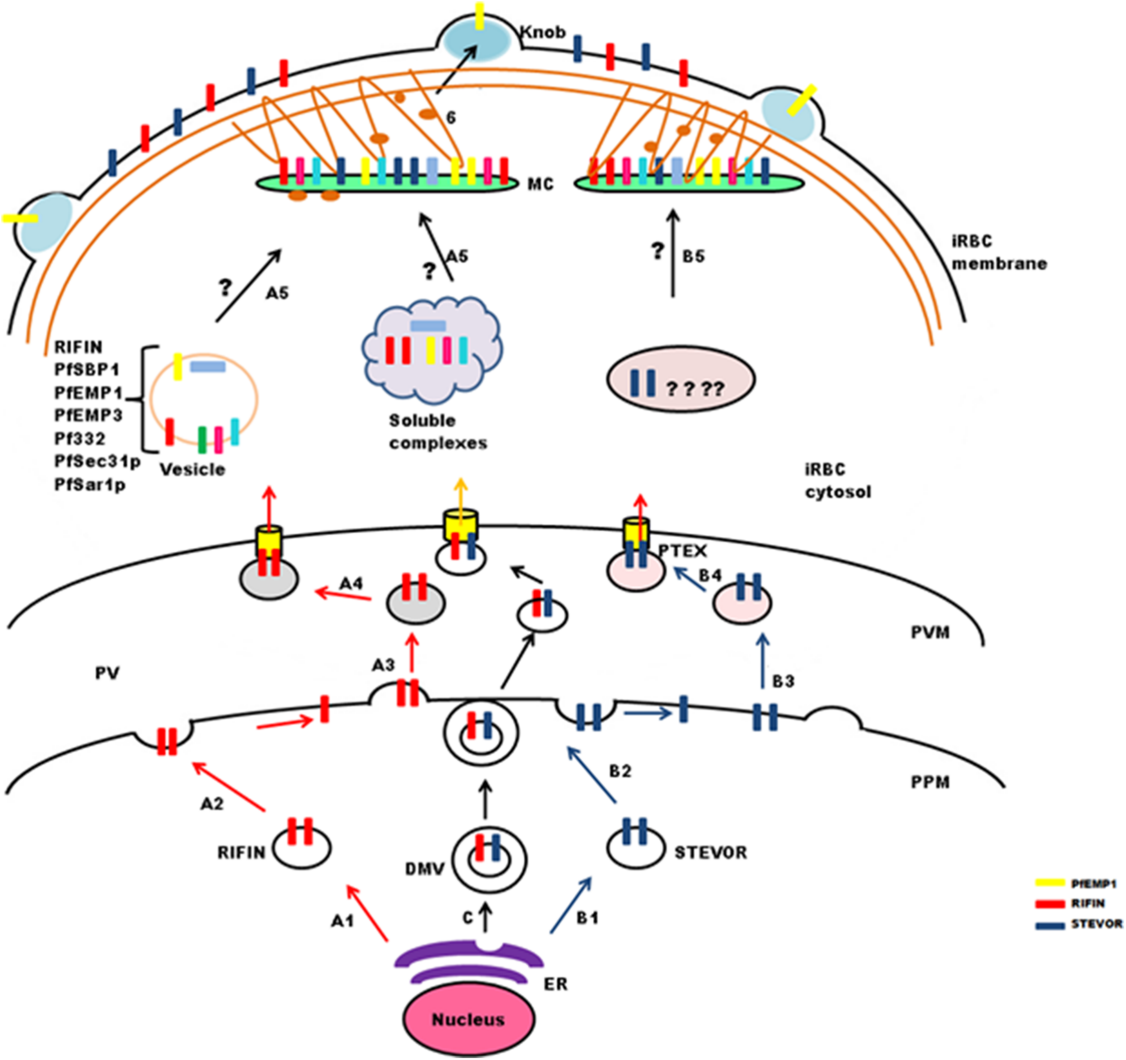
Schematic representation of possible trafficking pathways for STEVOR and RIFIN proteins. After synthesis and processing in the parasite ER budding vesicles are generated (StepA1 and B1), that fuse with the parasite plasma membrane (PPM) (Step A2 and B2), new vesicles are released (StepA3 and B3), that deliver proteins to the PTEX for export across the parasitophorous vacuole membrane (PVM) (StepA4 and B4). Trafficking may occur in the form of double membrane vesicles (DMVs) (C). RIFIN proteins are transported to Maurer’s cleft either in the form of vesicles or soluble protein complexes (StepA5), while the exact pathway for STEVORs remains elusive (StepB5), Proteins transiently localize to MCs before they are transported to the iRBC membrane possibly by vesicles treadmilling on actin filaments (Step6). Brown threads represent actin filaments.

Onward export of PlasmepsinV cleaved proteins from the ER may occur as trafficking cargo in the form of vesicles (Römisch, 2005) (Fig. 4: stepA1 and B1). These vesicles fuse with the PPM (Fig. 4: stepA2 and B2) where new vesicles are generated (Fig. 4: stepA3 and B3) that travel and bind to a specific region of the PVM having the *Plasmodium* translocon of exported proteins (PTEX) (Fig. 4: stepA4 and B4) that recognizes processed PEXEL residues (de Koning-Ward et al., 2009). Alternatively, double membraned vesicles formed by invagination of ER may also serve as a means of protein transport across the PPM (Fig. 4: step C1). In this case, proteins remain integrated in the inner membrane of DMVs. At the PPM, the outer membrane of DMVs and parasite membrane appose and fuse with each other (Fig. 4: step C2) leading to release of the inner vesicles that fuse with PTEX (Fig. 4: step C3). PTEX is a macromolecular complex that resides at the PVM, and is essential for transport of parasite encoded proteins across this membrane (Elsworth et al., 2014). Exported cargo includes both soluble and TM proteins carrying PEXEL, or even PEXEL negative exported proteins. PTEX consists of five components: EXP2, PTEX88, PTEX150, HSP101 and TRX2, which are proposed to play different roles in protein export (Elsworth et al., 2014). HSP101 forms a hexameric ring structure and carries ATPase activity that provides energy required for protein translocation. EXP2 forms pores traversing the membrane, and TRX2 helps in protein unfolding for translocation. While PTEX150 is putatively involved in maintaining the structural stability of the translocon, PTEX88 is a likely contributor in mediating parasite sequestration during in vivo growth.

### Transport from PVM to MCs and iRBCs

Maurer’s clefts form a continuous membranous network in the host cell cytoplasm, and carry markers different from those of the TVN (Haldar et al., 2005; Tamez et al., 2008). Translocated cargo proteins are either transferred to nascent MCs or delivered via small budding vesicles (McMillan et al., 2013). Synthesis of nascent MCs occurs ∼2 hpi (Grüring et al., 2011), while peak transcription of RIFIN and STEVOR proteins is observed between 12 and 24 hpi. Therefore, it seems likely that these proteins may either be trafficked in vesicles as a complex with other proteins to preformed MCs (Haeggström et al., 2004) or in soluble complexes (Papakrivos, Newbold & Lingelbach, 2005) like PfEMP1(Fig. 4: stepA5). Vesicular transport studies for RIFINs were carried out on *P. falciparum* field isolates and cultured parasites using antibodies specific for the conserved C-terminal region of PF3D7_0223100 (Haeggström et al., 2004). IFAs showed single small vesicles (SSVs) of ≤0.3 μm diameter on the trans side of PVM at 6–10 hpi, large multimeric vesicles (LMVs) of 0.1–10 μm diameter in the host cytosol at the trophozoite stage and large spindle like vesicles (LSLV) at >24 hpi beneath the iRBC plasma membrane. An identical pattern of budding vesicles was observed for PfEMP1 using antibodies specific for their conserved C-terminal acidic terminal segment or semiconserved N-terminal Duffy-binding-like-1 domain. Export kinetics of RIFINs suggest their cotransport with PfEMP1 from the PVM in the form of SSVs. This translocation is carried out by SSVs and LMVs at 12 hpi, LMVs at 16 hpi and LMVs as well as LSLVs at 20–28 hpi. MC marker protein Pf332 is also transported by LMVs, but spatially distributed to different subcompartments within these vesicles. PfEMP1/RIFIN loaded SSVs may fuse with transport machinery (PfSec31P, PfSar1P) to form the trafficking complex (LMVs) and subsequently LSLVs. The trafficking complex may include a number of other cargo proteins like Pf332, PfEMP3 and PfSBP1.

PfPTP1 is an integral membrane protein involved in maintaining the MC architecture by organizing actin filaments (Rug et al., 2014). Membrane topological studies show that its N-terminal region points within the MC lumen, and its C-terminal region lies in the erythrocyte cytosol. It colocalizes with PfSBP1 in MCs, but occupies different subcompartments here. PfPTP1 knock out parasite lines (CS2ΔPFPTP1) show accumulation of STEVORs in the PV suggestive of its role in transport of STEVORs across the PVM. Detailed mechanism about export of STEVORs from PVM to MCs is not elucidated (Fig. 4: stepB5).

Information regarding export of RIFINs or STEVORs from MCs to the iRBC surface is also not available, while PfEMP1 containing cargo vesicles that bud from MCs are believed to treadmill along actin filaments to reach the erythrocyte membrane (Cyrklaff et al., 2011, 2012; McMillan et al., 2013). Since the expression and export timings of PfEMP1 and RIFIN (PF3D7_0223100) overlap, it is probable that an identical process may mediate RIFIN trafficking (Fig. 4: step6).

## 2TM Proteins as Drug Targets and Vaccine Candidate

Repetitive interspersed family and subtelomeric variable open reading frames are surface exposed antigenically diverse protein families whose members are involved in immune evasion and rosetting. Immune evasion is an important strategy for survival of *Plasmodium* infected cells, conferring molecules implicated in this process the potential to serve as vaccine and/or drug targets. The phenomenon of rosetting is proposed to contribute to pathogenesis of severe malaria (Carlson et al., 1990; Kaul et al., 1991; Rowe et al., 1995; Wahlgren et al., 1992), and molecules that inhibit its occurrence could principally be developed for adjunct therapy of the disease. Candidates may be identified amongst the RIFINs and STEVORs for such therapies owing to the ability of members of these families to form rosettes independently of PfEMP1 (Yam et al., 2017). Though natural antibodies against STEVOR proteins have been found in plasma of patients, not enough information is available to explain host immune response against this family.

A subset of RIFIN proteins serve as ligands for inhibitory receptors LILRB1 or LAIR1 to mediate immune evasion by infected erythrocytes and suppression of host immune response (Saito et al., 2017). Binding extent of LILRB1 to host cell surface expressed RIFINs in malaria patients (small sample size) also shows direct correlation with disease severity. The presence of anti-RIFIN antibodies in sera of malaria infected individuals, their correlation with rapid parasite clearance and long term persistence (upto ∼ two years) suggests a protective role for these antibodies (Abdel-Latif et al., 2003). Broadly reactive antibodies that have incorporated the LAIR1 domain capable of binding to specific subsets of RIFINs and cause agglutination and opsonization of iRBCs have also been identified (Tan et al., 2016). Together, these findings highlight the RIFIN proteins as promising vaccine candidates and drug targets. Since RIFINs express differentially, are clonally variant and perform antigenic switching, target identification would require extensive transcript and proteome analysis. Additionally, structural and functional data on the individual members of this family is limited. Owing to their multistage expression and roles in immune evasion and suppression, members of the RIFIN family seem to be attractive components of multisubunit and multistage vaccines along with PfEMP1, PfMSP1 and circumsporozoite protein1. Therefore, it is important to either identify a strongly immunogenic member that is expressed at all parasite stages or design a consensus sequence based on surface exposed conserved epitopic regions of these proteins to generate broadly reactive antibodies against this family. Besides, detailed information on regions of RIFINs interacting with LILRB1 and LAIR1 may form the basis for rational drug and vaccine design. Post target recognition, generic challenges including determination of immunogenicity, large scale production of conformationally correct recombinant antigen, persistence of host response etc. shall need to be addressed. Since orthologues of these proteins are present in simian infecting parasites, chimpanzees and gorillas may be developed as potential animal models for vaccine studies.

## Conclusion

A comprehensive understanding of the functions of multigene families is necessary to completely uncover the link between parasite-induced pathology and antigenic variation on the surface of iRBCs. The highly diverse and clonally variant “2TM superfamily” is constituted of small variant surface antigen families, i.e., RIFINs, STEVORs and PfMC-2TM. This superfamily carrying ∼200 members is sparsely studied, with the role of PfMC-2TM family being a black box in parasite biology. Orthologues of the 2TM superfamily members are present in several simian infecting *Plasmodium* species, but absent from any of the other human, rodent or avian infecting *Plasmodia*. Interestingly, RIFINs and STEVORs share common secondary structural features and distant ancestry with the PIR superfamily comprising member families from *P. vivax* (VIR), *P. cynomolgi* (CIR), *P. knowlesi* (KIR), *P. berghei* (BIR) and *P. yoelii* (YIR) suggesting functional convergence in terms of generating antigenic diversity and evasion of host immune response. Information on RIFIN and STEVOR families is growing to reveal that these proteins are displayed on the surface of RBCs, and are involved in rosetting independently of PfEMP1. They are expressed at multiple stages of the parasite life cycle, and alter the mechanical properties of the host cell. Additionally, some of the members play a role in merozoite invasion contributing to malaria pathogenesis. Recent reports depict a clear role for some RIFIN proteins in immune evasion and host immune suppression, though the significance of STEVORs in generation of the host immune response remains obscure. Discoveries about interaction of RIFINs with LILRB1 and LAIR1 inhibitory receptors to promote parasite survival and suppress host immune effector molecules are likely to be important landmarks in completely unraveling malaria pathogenesis.

Apart from the B-type RIFINs, all studied members of the 2TM superfamily reside at the iRBC surface. In the absence of intrinsic host cellular components for export of parasite proteins, *Plasmodium* has devised its own translocation machinery for protein transport. RIFINs and STEVORs are PEXEL positive TM proteins that are trafficked through the classical secretory pathway within the parasite confines. This is followed by their translocation either as soluble protein complexes or in vesicles from the PVM to MCs. Beyond this step, export to the iRBC surface may be vesicle mediated. Notably, RIFIN export to the infected erythrocyte surface is spatially and temporally similar to that of the PfEMP1 family.

The 2TM superfamily lacks human homologues, and its members are emerging to be significant in disease pathogenesis. Correlation of rosetting with disease severity highlights molecules like RIFINs and STEVORs as prospective drug targets. Involvement of RIFIN proteins in key parasite processes like rosetting, immune evasion and modulation alongside their multistage expression underscores their potential as vaccine candidates. Identification of surface exposed members expressed at multiple parasite life cycle stages or common epitopes representative of most A-type RIFINs may lay the foundation for development of newer antimalarial vaccines. However, owing to antigenic diversity and clonal variation, drug or vaccine design against members of these families is expected to be quite challenging.

## Additional Information and Declarations

### Data Availability

The following information was supplied regarding data availability: The research in this article did not generate any data or code because this is a literature review article.
